# Reports of past alcohol and drug use following participation in a motivation enhancing intervention: Implications for clinical assessment and program evaluation

**DOI:** 10.1186/1747-597X-7-19

**Published:** 2012-05-14

**Authors:** David B Rosengren, Blair Beadnell, Mark Nason, Pamela A Stafford, Ray Daugherty

**Affiliations:** 1 Prevention Research Institute, 841 Corporate Dr., Suite 300, Lexington, KY, 40503, USA; 2 Alcohol and Drug Abuse Institute, University of Washington, 1107 NE 45th Street, Suite 120, Seattle, WA, 98105, USA; 3 School of Social Work, University of Washington, 4101 15th Ave NE, Seattle, WA, 98105, USA

**Keywords:** Self-report, Alcohol abuse, Alcohol dependence, Drug abuse, Drug dependence, Intervention, Motivation, Prevention, Assessment

## Abstract

**Background:**

There is significant interest in the value of motivational approaches that enhance participant readiness to change, but less is known about clients’ self-reports of problematic behavior when participating in such interventions.

**Methods:**

We examined whether participants in a motivationally-based intervention for DUI offenders changed their reports of substance use at postintervention (when reporting on the same 30 days that they reported on at preintervention). Specifically, Study 1 (*N* = 8,387) tested whether participants in PRIME For Life (PFL) changed their reports about baseline substance levels when asked at postintervention versus at preintervention. Study 2 (*N* = 192) compared changes in self-reported baseline drinking between PFL and intervention as usual (IAU) participants.

**Results:**

Many participants in Study 1 did not change their reports about how much they used substances during the 30-day period before baseline. Among those who did, the most common change was an increase in reported amounts of baseline drug use, and typical and peak alcohol use. This sample also showed changes in reports of their baseline pattern of high-risk-use (consistent versus occasional). At postintervention, participants who were younger, single, or endorsing more indicators of alcohol dependence were more likely to later report greater frequency of baseline drug use, and greater peak and typical number of baseline drinks. Gender, education, and race were also associated with reporting inconsistency on some behaviors. In Study 2, PFL participants showed greater increases in reports of peak alcohol use compared to IAU, but both conditions showed similar increases for drugs and typical alcohol use.

**Conclusions:**

In both research and clinical settings, a segment of participants may initially report less substance use than they do when asked later about the same baseline period. These preliminary findings suggest clinicians and researchers may find postintervention evaluations yield reports of greater baseline alcohol or drug use for some people. For some behaviors, this may occur more often in interventions that target client motivation. Future research should attempt to identify which reports – preintervention vs. postintervention – better reflect actual baseline substance use.

## Background

In clinical and program evaluation settings, self-report remains the most widely used approach for assessing socially sensitive behaviors [1-3]. Despite its widespread use, questions exist about the willingness of respondents to endorse socially undesirable behavior in self-reports [2,4]. While relevant across many behaviors, this concern applies to drug and alcohol abuse, its treatment, and research evaluation of intervention programs [5].

Del Boca and Darkes [6] describe three broad domains that can affect self-reports of alcohol and drug use: social context, respondent characteristics, and task attributes. Social context includes general considerations such as societal views and subcultural norms about substance use, as well as more specific issues such as the setting in which an assessment occurs. Respondent characteristics include demographic characteristics (e.g., age, race, ethnicity, and gender), reference groups (e.g., fraternities, religious organizations), personal characteristics (attitudes, beliefs and values), and circumstances (e.g., legal entanglements). In addition, respondent characteristics include difficulties in reconstructing past behavior (e.g., memory distortion or challenges) and transient factors such as intoxication, affective states, and physical condition. Finally, task attributes reflect the nature of the evaluation itself, such as mode of administration, task length, complexity of instruments, and formats. Del Boca and Darkes [6] assert social desirability is an underlying theme to these three domains. There have been efforts to address the effects of social desirability as a way to increase client disclosure about possible use and consequences, though it appears that indirect methods (e.g., Substance Abuse Subtle Screening Inventory – SASSI [7]) are no more effective than direct inquiries (e.g., Alcohol Use Disorders Identification Test – AUDIT [8]) [9,10].

A growing literature suggests methods to address these domains [6,10-13]. For example, Connors and Maisto [14] note being substance free and providing confidentiality increases the veracity of self-report. Del Boca and Darkes [13] add that evaluators should provide privacy, minimize assessment fatigue and boredom, and vary the assessment approach (e.g., use of an interviewer versus self-report) to enhance reporting. They also encourage use of real time data collection methods and collecting data from independent sources (e.g., collaterals or biological markers). While these methods may be employed in clinical and program evaluation settings, they are often difficult and sometimes impossible to implement. For example, community providers are often asked by courts to complete a drug and alcohol evaluation. Within that context, complete confidentiality of assessment results may not be possible. These challenges appear to contribute to less reporting of drug and alcohol use [15-17].

Stinchfield [18] looked beyond the assessment context itself and raised the question as to whether people’s self-report might change during participation in an intervention, even when being asked again about the same time period. His rationale hinged on clinicians’ observations that many clients report higher baseline levels of substance use after being in treatment for a period of time. To examine this, he assessed self-reported behavior during a highly structured Alcoholics Anonymous (AA) based intervention. In this study, 197 adolescents received inpatient treatment for alcohol and drug dependence. Participants reported on their baseline alcohol and drug use at pretreatment and reported again at discharge. Even though they reported on the same baseline period of time at discharge, participants described higher alcohol and drug use than they had at pretreatment. The greatest increase was in reports of alcohol use; 76% of the sample reported greater frequency of use when asked at posttreatment. Stinchfield [18] cautions against assuming that posttreatment reports were more accurate as there is no “gold standard” for comparison, though he does note that this is one potential explanation for his findings.

### Best practices for increasing cooperation and reports of alcohol and drug use

If reporting of use may change during intervention, might this be more likely with some counseling approaches than others? For example, might this be more likely with interventions that enhance motivation for change while decreasing defensiveness and resistance? Such intervention approaches exist, and there is an expanding research base that suggests that they can enhance motivation across a range of behaviors [19-22], including substance use services [23-28]. What is unknown is the extent to which participants in such interventions may alter their reporting on substance use such that they describe different amounts or patterns of drinking, even when reporting on the same period of time and to the same questions.

These approaches rely on conceptual frameworks such as the Transtheoretical Model [29], and clinical methods like motivational interviewing [30] which propose that motivation is amenable to influence. Moreover, these frameworks attempt to reduce resistance, which prior research indicates may increase client willingness to discuss problematic behavior [31]. Some indicated prevention programs, such as PRIME For Life (PFL) [32], share these foundations.

PFL is a theory-based, indicated prevention program that focuses on altering substance use-related risk awareness and intrinsic motivation for change. Widely employed in the U.S. for court-ordered DUI offenders, PFL uses the Transtheoretical Model [33,34] to understand how and why change occurs, builds on motivational interviewing (MI) concepts of relationship and technical components in content delivery [35], and uses MI and persuasion theory [31,36] in meeting resistance and gently challenging common views about risk for developing alcoholism and drug addiction. PFL curriculum also aims to help clients learn about the universal risk for alcohol- and drug-related problems, examine factors that influence risk and the development of alcohol and drug problems, and self-assess personal risk for problems. Based on their conclusions, participants develop an appropriate plan for protecting things they value. By combining this curriculum and MI elements, including a focus on the instructor-client relationship and client language consistent with MI principles, PFL seeks to enhance motivation and improve client outcomes in the context of a group intervention.

Despite the apparent logic that self-reports of socially undesirable behavior—even when asked again about the same period of time—might change, there are no published reports on this in the context of motivational interventions. It is particularly important that any such evaluation have sufficient power to evaluate whether other factors (e.g., demographics, risk factors) are associated with these types of changes in reporting, as suggested by Del Boca and Darkes [6].

### Study purpose

This investigation utilized two studies to compare preintervention to postintervention reports of the amount of substance use that occurred in the 30 days prior to baseline. Study 1 examined changes in self-report that occurred among participants in a motivationally based intervention (PFL). The primary hypothesis was that participants would report that they had used greater amounts of drugs and alcohol in the 30 days before baseline when asked following intervention compared to when asked at preintervention. We then utilized the strength of this large sample to perform analyses to answer four exploratory questions: (1) what percent of the sample remained consistent versus changed their reports; (2) among those who changed, how many increased versus decreased the amount of baseline use they reported and what was the magnitude of the change, (3) was change in self-report related to participant characteristics, and (4) what changes occurred in baseline drinking pattern characterizations (e.g., occasional versus consistent high-risk drinkers)?

Study 2 tested whether participants in an intervention that explicitly and in a structured manner targets motivation (PFL), compared to one that does not (IAU), showed differential changes in postintervention reports of baseline use. The hypothesis was that following the motivational intervention, participants would report greater baseline substance use compared to participants in the IAU.

## Methods

### Overview

This secondary analysis used de-identified data from existing program evaluations of PFL. We submitted the study methods for human subjects review to Western IRB (WIRB) who determined that use of this de-identified data was exempt from the requirement for IRB review under 45 CFR §46.101(b)(4). Participation in the program evaluation was voluntary.

### Samples

Two samples provided data for analysis. The Study 1 sample (*N* = 8,387) consisted of participants from eight states (GA, IA, IN, ME, NC, ND, SC, and UT) convicted of impaired driving or other alcohol- or drug-related offenses who received PFL from 2006 to 2008. Most participants had been referred for impaired driving. In each of the eight states, they attended a PFL program provided by alcohol/drug DUI (or its state-specific equivalent) schools or treatment agencies. Completion of the course was typically a prerequisite for reinstatement of driving privileges.

The Study 2 sample (*N* = 192) included the North Carolina participants from the Study 1 sample and additional individuals from North Carolina who attended a non-PFL intervention program. This intervention was the standard in North Carolina prior to the implementation of PFL statewide and the study occurred during the period when the state was crossing over between these two programs. We refer to this non-PFL program as Intervention as Usual (IAU). Data collection for the PFL and IAU samples occurred concurrently in 2007–2008. Because participants took part in a class based on their schedule and class availability, assignment was not random. To ensure equivalency between the two conditions, we performed a post hoc matching. For this, we selected a subset of participants from the larger PFL group that were matched to IAU participants in a 2:1 ratio (*n* = 128 and 64, respectively). Using a macro generated in SAS [37], we created propensity scores – based on preintervention demographics (age, education, and race) and substance use (prior 30-day drinking and drug use) – to match each IAU participant with two PFL individuals sharing similar characteristics. As shown in the Sample Description section (below), the resulting sample contained groups that were similar on gender, marital status, age, education, race, and substance use.

### Procedures for study 1 and 2 samples

Instructors received structured protocols and scripts for all procedures. They performed all data collection procedures, including describing the study, distributing the questionnaires at both the preintervention and postintervention time points, and mailing these questionnaires to the research staff. All measures were self-administered, paper and pencil questionnaires. Instructors offered to administer the questionnaires to participants who preferred having the questions read.

#### Recruitment and enrollment

At the beginning of the first class, instructors informed participants of the purpose of the evaluation, that responses were anonymous, and that participation was voluntary. Then, they distributed the questionnaires. All participants received copies of the assessment instrument, but could refuse participation by not completing them.

#### Data collection

Participants filled out the questionnaires before the first class began, and then immediately after the last class ended. Because curriculum schedules varied considerably between states, the length of time between the preintervention and postintervention questionnaires ranged from 3 days to 8 weeks. When participants first arrived they received two matched preprinted labels, which they adhered to their program materials. They affixed the first preprinted label to the preintervention questionnaire immediately. The second label was kept and then affixed to the postintervention questionnaire at the time of that assessment. This procedure allowed participant questionnaires to be matched while maintaining their anonymity. At both preintervention and postintervention, participants placed their questionnaires into an envelope, which was then sealed by the last participant. The instructor then mailed these sealed, stamped, and self-addressed envelopes to Prevention Research Institute (PRI) for data entry and analysis. All questionnaires – completed or not – were returned to PRI. These procedures prevented instructor knowledge of responses.

### Measures

The self-report, paper and pencil measures took participants approximately 15 min to complete. Except for demographic items, all pretest questions were repeated at postintervention. All items had been pilot-tested in prior program evaluations.

#### Substance use

Questions concerning quantity of drinking and frequency of marijuana or other drug use targeted the 30 days prior to program participation. We asked these questions covering the same 30-day preintervention period on both the preintervention and postintervention questionnaires. The questions came from epidemiological studies that used graduated frequencies to assess patterns and peak use amounts [38,39], while building on factors that prior research indicates increase reliability and validity of alcohol and drug reports [38]. The substance use response categories allowed us to differentiate people who were abstainers (0 drinks), low-risk drinkers (1–3 drinks), and high-risk drinkers (4 or more drinks), a categorization scheme based on the low- and high-risk guidelines taught in the PFL program. Drug use categories allowed us to identify the continuum of use, but also to differentiate use versus nonuse, as the PFL guidelines regard zero as the only low-risk option for drug use (due to the risk of impairment-related problems).

For peak amounts of drinking, participants indicated the most drinks they had consumed in a single day during the 30 days prior to their participation in the program; they also reported usual number of drinks in a day for the same period. Response categories were 0, 1–3, 4–6, 7–9, 10–12, 13–15, 16–18, 19–21, 22–24, and 25 or more. PRI asked instructors to teach the definition of a standard drink prior to participants answering the drinking questions. A similar item asked about marijuana/other drug use with six response categories (0 = never, 1 = 1 time, 2 = 2–3 times, 3 = about once a week, 4 = 2–3 times a week, and 5 = most days). Due to sparse distributions, we combined some response categories for analyses purposes. Specifically, we collapsed responses for upper ranges in peak number of drinks into a single category of 13+ drinks and usual number of drinks to 10+ drinks. For marijuana or other drug use, we combined responses to form three categories (0 = never, 1 = once a week or less, and 2 = more than once a week). Prior pilot tests show test-retest correlations of .91 for typical drinks, .89 for maximum number of drinks and .73 for drug use.

#### Indicators of possible alcohol dependence

At postintervention, we used six items as indicators of alcohol dependence symptoms drawn from the Alcohol Use Disorders Identification Test (AUDIT) and from Russell and colleagues analyses of the Health Interview Scale [8,40]. These items included such statements as “Have you sometimes taken a drink in the morning when you first got up?” and “During the last year have you failed to do what was normally expected of you because of your drinking?” Response categories were yes (= 1) and no (= 0). We categorized participants as having 0, 1 to 2, or 3 to 6 indicators.

### Drinker type

At preintervention and postintervention, we categorized participants into three drinking groups, each representing a pattern of drinking based on PFL low-risk drinking guidelines. These guidelines stem from an extensive review of the health and impairment consequences associated with use levels [32]. These recommend participants drink no more than one drink per hour, average no more than two drinks per day across a week, and consume no more than three drinks on any drinking occasion. Accordingly, we categorized participants who either did not report drinking or reported always consuming three or fewer drinks on days they drank as Low-Risk (LR) drinkers. We developed two additional risk categories as a way to differentiate risky drinkers based on the conceptual idea that some people drink in high-risk amounts whenever they drink, and others only do so occasionally (e.g., weekends). This categorization separated individuals into a group whose typical use was high risk, and the others into a group whose typical use was low risk but who did at times engage in high-risk use. Participants who reported typically drinking three or fewer drinks but consuming more than that at least once within the 30 days prior to the program were categorized as Occasional High Risk (OHR) drinkers. Those who reported typically consuming more than three drinks per drinking day were categorized as Consistent High-Risk (CHR) drinkers.

It should be noted that because we did not have frequency data, there is the possibility that some participants in the LR category drank three drinks daily or near daily. This level of drinking would place them in a high-risk category within the PFL guidelines used in these analyses. However, for the purposes of this paper, this group still appears distinct from the other two categories of drinkers, who were drinking four or more drinks at least once a month or more.

### Intervention conditions

While there were curriculum differences, a key distinction in PFL versus the IAU is the integration and standardization of motivational techniques. While IAU included recommendations to address motivation and to use motivational interviewing techniques, it did not explicitly integrate this into the curriculum, session structure, or program delivery; PFL did.

#### PRIME for life

PFL is a theory-based program designed to enhance participant motivation for changes while exploring high-risk alcohol and drug choices. It is not an abstinence-only based program, but instead focuses on low-risk choices. Such choices may include abstinence for some but not all individuals. PFL places a strong emphasis on the manner in which the intervention is delivered since there is empirical support for the value of such process variables in the delivery of substance abuse interventions [30,41]. Specifically, PFL employs three elements of empirically-supported practices for alcohol and drug use interventions: a) establishing a collaboration with participants, b) diffusion of resistance and c) a clear direction on the part of the interventionist [30,42]. The Lifestyle Risk Reduction Model [43,44], the Transtheoretical Model [45], and persuasion theory [46,47] guide the progress and activities of the PFL intervention.

PFL is manual guided and structured. Program length varied between 12 and 20 h, depending on state requirements. The standardized curricula designed for each of these lengths contains the core program elements. In NC, PFL occurred in 16-h groups, typically over a two-day period. Other locations could have massed (e.g. three sessions over the course of a weekend) or shorter, distributed meetings (e.g., two hour sessions over an eight week period).

#### Intervention as usual

In NC, the IAU curriculum was also manual-based, lasted 16 h, and included a list of substance abuse topics and presentation guidelines. Instructors had significant flexibility in choosing session topics. The program encouraged use of motivation-based techniques, but their use was not standardized, intensively trained, nor required. Instructors made content choices based on salience for particular groups. Possibilities included information about impaired driving laws, the scope and problems of driving impaired, the physical effects and historical perspective of alcohol and drug use (concepts of use and abuse, the disease concept, information on special populations), and assessing personal issues (examining own use, financial costs of an impaired driving arrest, identifying personal problems related to use, life skills, and available treatment options). Resources and handouts supplemented this content.

### Statistical approach

For the Study 1 analysis of repeated measures, we used Generalized Estimating Equation (GEE; [48]) in PASW 18.0. GEE was well-suited to these analyses in that it allowed specification of different covariance structures, calculated robust standard errors, and allowed the analysis of ordinally measured outcomes. We also used Chi-square tests to compare patterns of reporting (reporting less, the same, or more at postintervention compared to preintervention) with participant characteristics. For the Study 2 analysis of repeated measures, we used mixed effects regression using PROC GLIMMIX in SAS 9.2 [49]. This has the same advantages of GEE but also allowed us to adjust results to account for the non-independence introduced into the data by the Study 2 matching procedures described earlier. All other analyses were descriptive.

## Results

### Sample descriptions

The Study 1 sample (*N* = 8,387) was composed of more males (76%) than females (24%). The majority of participants were white (88%), with the remaining participants being African American (5%), Hispanic (3%), or other race/ethnicity (4%). The mean age was 32 years (*SD* = 11.85). The majority of participants had received either a technical school or college degree (86%) and had never been married (51%). Approximately 29% of participants reported zero substance dependence indicators with the remainder reporting one to two (38%), or three to six (33%).

The Study 2 sample (*N* = 192) also consisted of more males (64%) than females (36%). Participants were primarily white (87%) with the remainder being African American (6%) or other people of color (7%). Mean age was 32 (*SD* = 11.41). Most participants had never married (53%), but had obtained at least a technical school degree (77%). One-third (33%) of participants reported zero substance dependence indicators with the remaining participants reporting either one to two (46%) or three to six indicators (21%). The matching procedure was successful in balancing the PFL and IAU groups on demographic variables. For example, PFL versus IAU samples were 35% vs. 38% females, 86% vs. 88% white, 52% vs. 58% never married, and 78% vs. 77% having at least a technical degree. Mean age for PFL vs. IAU was 32 vs. 33, and mean number of indicators of dependence was 1.5 vs. 1.2. Preintervention means on the substance use variables were also balanced across conditions.

### Study 1: Self-report change during PFL

#### Preliminary analysis

Because of the variability in time between preintervention and postintervention across states in Study 1, we examined whether the results differed depending on the length of elapsed time. There were no discernable patterns and so we combined data across settings.

#### Change from preintervention to postintervention

As shown in Table 1, the Time effect in GEE analyses – which represents change from preintervention to postintervention – showed that participants overall changed the amount of baseline (the 30 days prior to intervention) substance use they reported. For all three outcomes (i.e., usual number of drinks in a day, peak number of drinks in a day, and marijuana or other drug use), more participants reported higher levels of baseline use at postintervention. For both types of drinking, fewer participants reported baseline use in the zero and one-to-three drink range postintervention than they had at preintervention. For most drinks in a day (peak drinking), a greater proportion of the sample appeared in the highest (7–9, 10–12, and 13+) categories. For usual number of drinks, more participants appeared in the 4–6, 7–9, and 10+ categories. With regard to marijuana and other drug use, more of the participants reported baseline use of once a week or more (with fewer reporting abstinence) at postintervention than they had at preintervention.

**Table 1:** Preintervention versus postintervention comparisons of PFL participants’ reports of their substance use in the 30 days before preintervention

	**Percent**		**Time**^**3**^	
	**Preintervention**	**Postintervention**	**Wald *χ***^**2**^	***p***
			**(*df*** **=** **1)**	
Peak drinks^1^			656.37	<.001
0 drinks	35.3%	28.9%		
1-3 drinks	22.9%	17.7%		
4-6 drinks	17.2%	17.2%		
7-9 drinks	9.1%	11.8%		
10-12 drinks	7.0%	9.6%		
13+ drinks	8.4%	14.8%		
Usual drinks^1^			615.34	<.001
0 drinks	34.8%	27.8%		
1-3 drinks	35.9%	30.0%		
4-6 drinks	17.1%	22.0%		
7-9 drinks	6.2%	10.1%		
10+ drinks	6.0%	10.1%		
Marijuana or other drug use^2^			317.90	<.001
Never	85.5%	78.7%		
1x/week or less	7.3%	11.1%		
More than 1x week	7.2%	10.2%		

^1^ *N* = 8,387.

^2^ *N* = 8,366.

^3^Estimated from GEE analysis.

#### Patterns of change

**Figure 1:**
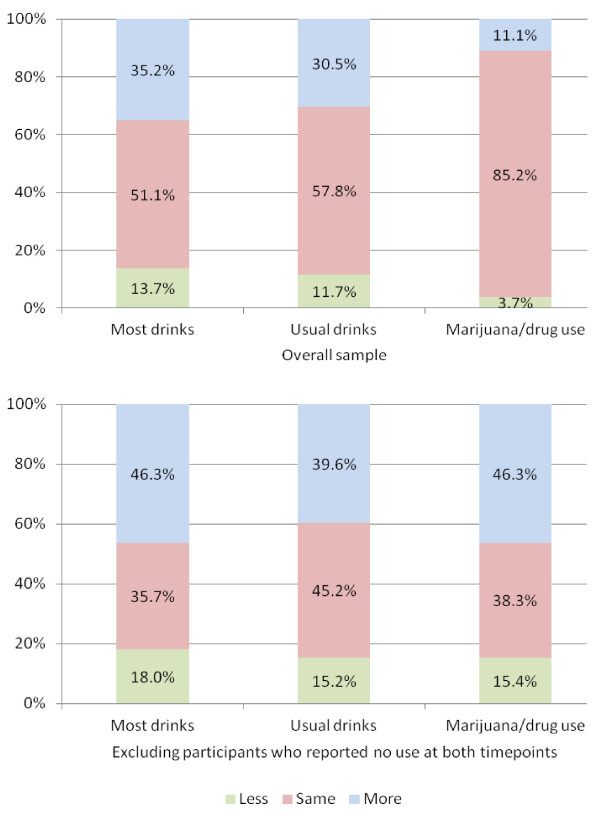
Percent of participants who at postintervention reported more, less, or the same substance use amounts than they did at baseline (for the 30 days before intervention).

Bolded percents (the diagonals) in Table 2 show the proportion of participants whose preintervention reports of use in the 30 days prior to baseline were consistent with their responses at postintervention. Below the diagonal are the percent reporting greater baseline use at preintervention compared to postintervention. Above the diagonal are those who reported greater baseline use at postintervention. The largest percentages of people who did not change were those in the lowest (no use) and highest drinking and drug use categories. The most common change was to the next highest category; however, there were many cases where participants changed to much higher reports at postintervention. Figure 1, which summarizes this data, shows that when change did occur, it was most often in the direction of reporting more baseline use. To focus more specifically on the reporting patterns of people who acknowledged some use of alcohol or drugs, we excluded people who may have been true abstainers (e.g., reported no use of the specific substance at both assessment time points). As shown in Figure 1, about half of these people reported more baseline peak drinking and drug use when asked at postintervention than they had preintervention, and about 40% did so for usual number of drinks.

**Table 2:** Percentage of postintervention response categories by preintervention response (For 30-Day period prior to intervention)

	**Preintervention**	**Postintervention**					
		**0 drinks**	**1-3 drinks**	**4-6 drinks**	**7-9 drinks**	**10-12 drinks**	**13+ drinks**
Peak number in a day	0 drinks	**68.1**	11.9	6.8	4.2	3.6	5.4
	(*n* = 2,963)						
	1-3 drinks	12.1	**42.5**	24.2	9.3	6.1	5.8
	(*n* = 1,923)						
	4-6 drinks	5.6	13.0	**38.9**	21.3	11.8	9.4
	(*n* = 1,443)						
	7-9 drinks	4.7	7.4	15.6	**32.5**	21.9	17.9
	(*n* = 761)						
	10-12 drinks	4.9	6.6	11.0	13.6	**27.2**	36.7
	(*n* = 589)						
	≥13 drinks	4.2	4.2	4.2	7.2	11.6	**68.6**
	(*n* = 708)						
		**0 drinks**	**1-3 drinks**	**4-6 drinks**	**7-9 drinks**	**10+ drinks**	
Usual number of drinks	0 drinks	**66.0**	15.7	9.6	4.2	4.5	
	(*n* = 2,918)						
	1-3 drinks	8.5	**56.9**	24.2	6.0	4.4	
	(*n* = 3,008)						
	4-6 drinks	6.6	17.9	**48.1**	17.7	9.7	
	(*n* = 1,438)						
	7-9 drinks	3.9	8.9	20.1	**40.7**	26.4	
	(*n* = 518)						
	≥10 drinks	5.9	8.9	9.3	15.5	**60.4**	
	(*n* = 505)						
		**Never**	**≤1x week**	**≥2x week**			
Per week marijuana drug use	Never	**88.8 **	7.4	3.8			
	(*n* = 7,121)						
	≤1x week	25.2	**53.2**	21.6			
	(*n* = 607)						
	≥2x week	12.7	13.2	74.1			
	(n = 598)						

Table notes:

Diagonal percents are bolded and represent participants who provided the same response at preintervention and postintervention; those under the diagonal represent reported greater use at preintervention and those over the diagonal reported greater use postintervention. All reports were about the 30 days prior to intervention.

#### Association of participant characteristics with patterns of change

Participant characteristics were associated with changes in reporting (Table 3). Specifically, there was a greater tendency to report more baseline use at postintervention among those who were younger, single, and had more dependence indicators. These three groups showed the same reporting pattern for peak number of drinks, usual number of drinks, and marijuana/other drug use. There were other characteristics associated with more specific issues in reporting. For example, people with college degrees were more likely than others to increase the peak number of baseline drinks reported, while those with some college education were more likely to endorse marijuana/other drug use. White participants were more likely to report more peak number of drinks. There were two instances where characteristics were associated with a tendency to report smaller amounts at postintervention: people of color and men did so for their usual number of drinks, though for men it was a small and perhaps not clinically meaningful difference.

**Table 3:** Association of PFL participant characteristics with changes in amount of substance use reported for the 30 days prior to intervention

		**Postintervention relative to preintervention**				
		**Less**	**Same**	**More**		
**Participant characteristic**	***n***	***%***	***%***	***%***	***χ***^***2***^***(df)***	***p***
**Peak number of drinks in a day**						
Age					48.79 (8)	<.001
<24	2,980	14.1	47.0	38.8		
25-34	2,332	12.7	52.0	35.3		
35-44	1,562	12.7	54.4	32.9		
45-54	1,082	14.5	54.3	31.2		
55+	427	16.9	55.5	27.6		
Education					28.30 (8)	<.001
<High school (n = 89)	89	12.4	59.6	28.1		
High School/GED	207	14.5	54.6	30.9		
Some college	653	18.8	50.2	30.9		
Tech school graduate	2,900	13.5	53.1	33.3		
College degree(s)	2,696	13.1	49.9	37.1		
Race					12.34 (2)	.002
White	7,280	13.3	50.8	35.9		
Non-white	1,007	16.0	53.2	30.8		
Marital Status					20.96 (4)	<.001
Never married	4,137	14.0	49.0	36.9		
Married/live together	2,290	12.3	52.9	34.8		
Divorced/separated/widowed	1,751	14.7	53.4	31.9		
Number of dependence indicators					140.91 (4)	<.001
0	2,410	14.8	59.3	26.0		
1-2	3,171	14.4	48.3	37.3		
3-6	2,793	11.9	47.3	40.8		
**Usual number of drinks**						
Gender					7.55 (2)	.023
Male	6,227	12.2	57.3	30.5		
Female	1,948	10.0	59.6	30.4		
Age					32.69 (8)	<.001
<24	2,980	11.5	55.1	33.5		
25-34	2,332	11.7	57.7	30.6		
35-44	1,562	10.7	59.7	29.6		
45-54	1,082	12.9	61.2	25.9		
55+	427	13.6	61.4	25.1		
Race					7.04 (2)	.030
White	7,280	11.3	58.0	30.7		
Non-white	1,007	14.1	56.4	29.5		
Marital Status					14.77 (4)	.005
Never married	4,137	11.8	56.1	32.1		
Married/live together	2,290	10.8	60.2	29.0		
Divorced/separated/widowed	1,751	12.7	58.7	28.6		
Number of dependence indicators					161.70 (4)	<.001
0	2,410	12.5	65.9	21.6		
1-2	3,171	12.0	56.9	31.1		
3-6	2,793	10.6	51.6	37.7		
**Marijuana or other drug use**						
Age					81.63 (8)	<.001
<24	2,965	4.5	80.9	14.6		
25-34	2,311	3.4	86.0	10.6		
35-44	1,547	3.1	88.3	8.6		
45-54	1,080	3.8	87.9	8.3		
55+	419	1.9	92.1	6.0		
Education					29.61 (8)	<.001
<High school	83	3.6	85.5	10.8		
High School/GED	203	2.0	86.2	11.8		
Some college	647	6.6	78.1	15.3		
Tech school graduate	2,877	3.2	84.7	12.2		
College degree(s)	2,687	4.4	84.3	11.3		
Marital Status					45.68 (4)	<.001
Never married	4,109	4.3	82.8	12.9		
Married/live together	2,278	2.6	87.8	9.6		
Divorced/separated/widowed	1,733	3.5	88.1	8.4		
Number of dependence indicators					70.61 (4)	<.001
0	2,397	3.7	88.2	8.1		
1-2	3,148	3.6	86.4	10.0		
3-6	2,768	3.8	81.1	15.1		

We did not include nonsignificant associations for gender with peak number of drinks and marijuana/other drug use (*χ*^*2*^ *=* 2.46*, df =* 2, *p* = .293 and *χ*^*2*^ *=* 2.99*, df =* 2, *p* = .225; respectively), education on usual number of drinks (*χ*^*2*^ *=* 7.71*, df =* 8, *p* = .463), or race on marijuana/other drug use (*χ*^*2*^ *=* 4.81*, df =* 2, *p* = .09).

#### Changes in drinker type categorization

Comparisons of drinker type assessed at preintervention versus postintervention revealed that nearly one-third (31.6%) of the sample changed their information enough between preintervention and postintervention that their baseline drinking group categorization changed. Specifically, 17.5% of the sample reported LR drinking patterns at preintervention but were subsequently identified as either an OHR or CHR drinker postintervention. Conversely, 4.9% of participants initially reported high-risk baseline drinking behavior (either OHR or CHR patterns) but were identified as LR drinkers postintervention. The remaining 9.2% of those whose risk categorization changed were identified at both preintervention and postintervention as a high-risk drinker (either OHR or CHR), but changed which high-risk group they were in at postintervention. Most often, people were categorized at preintervention as occasionally engaging in high-risk drinking (OHR, 6.5%) but at postintervention as doing so more consistently (CHR). A smaller number (2.7%) were categorized at preintervention as CHR but then as OHR at postintervention.

### Study 2: Comparison of change: PFL vs. IAU

The NC sample allowed for comparison of substance use reports across two intervention conditions – participants who received PFL and those who received intervention as usual (IAU). As shown in Table 4, PFL participants demonstrated greater change compared to IAU in their reports of peak number of drinks consumed in a day during the 30 days before the intervention (the Time X Group interaction). Specifically, PFL participants were less likely at postintervention to report that they had consumed zero or one to three drinks in the 30 days prior to baseline and more likely to report having consumed four or more drinks. In contrast, IAU participants showed virtually no change. In terms of the usual number of drinks and marijuana/drug use, both intervention groups demonstrated increases in reported baseline use, with no significant differences between the two.

**Table 4:** Changes in self-report of substance use in the 30 days prior to intervention for PFL versus IAU in North Carolina

	**Condition**				**Wald *χ***^**2**^**(*df*** **=** **1)**^**a**^			
	**IAU(*n*** **=** **64)**		**PFL(*n*** **=** **128)**		**Time**		**Time X group**	
	**Preintervention**	**Postintervention**	**Preintervention**	**Postintervention**	***F(df)***	***p***	***F(df)***	***p***
	**Peak number of drinks in a day**^**b**^							
0	20.3%	23.4%	15.5%	12.4%	2.29	.13	7.25	<.007
1-3	29.7%	31.3%	35.7%	20.2%	(1, 379)		(1, 379)	
≥4	50.0%	45.3%	48.8%	67.4%				
	**Usual number of drinks**^**b**^							
0	19.0%	14.3%	15.5%	11.6%	20.97	<.001	0.96	.330
1-3	58.8%	50.7%	67.4%	51.9%	(1, 191)		(1, 191)	
≥4	22.2%	35.0%	17.1%	36.5%				
**Per week marijuana/other drug use**								
Never	92.3%	83.1%	92.2%	86.0%	15.01	<.001	0.77	.313
≤1x	3.1%	9.2%	7.0%	11.7%	(1, 192)		(1, 192)	
≥2x	4.6%	7.7%	0.8%	2.3%				

Note: Group, Time, and Time X Group effects were estimated from GEE analysis.

^a^The Time and Time X Group effects were of primary interest. Group effects for peak number of drinks, usual number of drinks, and per week marijuana/other drug use: *F =* 2.43*, df =* 1,105, *p* = .122; *F =* 0.02*, df =* 1,192, *p* = .894; *F =* 1.02*, df =* 1,192, *p* = .313.

^b^For parsimony, the *4*–*6*, *7*–*9*, *10*–*12*, and *13+* drinks categories were collapsed into one category representing high risk drinking.

## Discussion

How to best assess drug and alcohol use and misuse has been a longstanding challenge. While methods that address assessment conditions can affect reporting, there is little known about whether clients change their reporting of alcohol and drug use (i.e., that is, report different amounts even when about the same period of time) when attending interventions focusing on client motivation. In examining this, we found that while many clients do not alter their reports of the amounts of alcohol and drugs they used in the 30 days prior to baseline, a noticeable segment do. When change occurred, the most common alteration we observed for alcohol use was reporting greater baseline amounts at postintervention. Some participants also reported greater baseline drug use, and while only a small percent of the overall sample made this change, a striking number (about half) of those who eventually reported using drugs in the 30 days before baseline had previously claimed abstinence for that period of time.

In terms of the difference between PFL and IAU, the reports of baseline drug use and usual number of drinks increased overall for both conditions, which suggests that receiving an intervention is more important than the type of intervention. Still, the motivational-based intervention appears to have led to greater reports of peak baseline use, which is intriguing as the PFL curriculum specifically targets the risks associated with high levels of peak consumption, even when done infrequently. However, we cannot rule out alternative explanations, such as another curriculum difference or greater adherence to a standardized protocol, being the cause of this difference. This question warrants future research about the nature and causes of these changes.

### Understanding changes in reporting

It is important to keep in mind what Stinchfield [18] points out: without a clear “gold standard” for comparison, it is not possible to conclude definitively which assessment time point is more accurate. This study did not include corroborative data (i.e., collateral reports, biomedical markers) which would have provided a basis for drawing conclusions about accuracy. Future research on this topic should attend to this important topic by collecting such information.

With that caveat, the findings bear speculation about why such changes occur in the reporting of a fairly concrete behavior. An obvious potential explanation for why a subset of people might increase their postintervention reports of their baseline behavior is that they became more open to describing their substance use. Underlying this study’s conceptualization was the possibility that this response might occur during a motivational intervention that focuses on decreasing resistance and defensiveness. This interpretation is plausible given that participants were experiencing legal consequences and required to take part in these interventions. In that context, it would not be surprising that they might initially be defensive, unlikely to view themselves as having substance use problems, and unwilling to fully disclose the extent of their use. This explanation warrants exploration in future research, since the current study did not measure defensiveness, so could not adequately test it as an explanatory mechanism.

Other obvious explanations for our findings exist and should be considered. One alternative is people misremember the amount of substance(s) used, but the intervention experience jogs their memory. Another is that since PFL contains an educational component about what constitutes a standard drink, improved understanding of this may cause a change in the number of drinks they report (although the questionnaire administration protocol directed instructors to teach the definition of a standard drink prior to participants answering the questionnaires). Overall, it is quite possible that each of these factors discussed here – defensiveness, memory, understanding of standard drinks -- play some role in the self-report inconsistencies we observed. It is important to note that all of these alternative interpretations imply that postintervention reports are more accurate than preintervention reports. However, it may be that the opposite is true. The data do not permit us to determine which is more accurate and so this needs to be a focus for future research. Such research might also profitably tease out the relative contributions of each of the explanations offered.

### Individual differences in self-report change

There were some participant characteristics related to changes in reports of baseline use. We observed that participants who were younger, single, and with more indicators of possible dependence were more likely to report greater baseline use at postintervention compared to their peers. There were also intriguing instances of patterns of greater postintervention reporting among whites and people with college degrees on peak number of drinks. Those with some college education were more likely to report greater drug use compared to people with either greater or lesser education. In contrast, people of color and men were more likely to report a smaller number of usual drinks at postintervention. Why these findings occurred is unclear. If it is the case that postintervention reports are more accurate, it may be that social desirability, as suggested by Del Boca and Darkes [6], is at work in these different circumstances though for different reasons. Younger, single substance users may want to view their use as part of a social circumstance that encourages youthful rebellion, while those with greater indicators of dependence may be engaged in a more internal, psychological defense against the mounting evidence of possible problems. However, these interpretations are speculative and further inquiry is needed.

### Implications

The findings address a common belief that individuals who use alcohol and drugs underreport their use, and that clinicians should typically adjust clients’ self-reports upward. Indeed, many participants did report higher use postintervention. However, it is important to note that the majority of participants were consistent in their reports and others reported less use following intervention. These findings, in combination with the unanswered question as to which report is most accurate argues against the idea of providing a “correction factor” to initial self-reports since that might lead to some individuals being referred to unneeded services.

These findings also have implications for research and program evaluation purposes. The between-condition comparisons in Study 2 indicated that PFL participants endorsed heavier peak drinking and both groups endorsed greater drug use and typical drinking amounts postintervention. Such inconsistencies could lead to invalid conclusions in evaluations. For example, if posttreatment reports were more accurate, our findings raise concern that program evaluations might underestimate the magnitude of intervention effects because of a tendency to later report greater use. For example, if participants report less use at preintervention but report more use at follow-up points, consumption may appear unchanged or even increased over time, when in actuality it has decreased. Moreover, it may be that conditions specifically targeting motivation may be at differential risk for this sort of Type II error, especially when compared to waiting list or non-motivational intervention controls.

Future research on these issues -- especially that which sheds light on whether later reports have greater accuracy – is important to inform policy related to the timing of assessments. Particularly with arrests for driving under the influence (DUIs), courts typically order assessments *prior* to assigning people to either treatment or indicated prevention. However, the wisdom of that approach depends on when the most accurate self-reports can be gathered. If preintervention reports are more trustworthy, then the typical approach is sensible. However, if postintervention reports have greater accuracy, it may be more useful if offenders are referred to a motivationally-based intervention first. A better understanding of when the information gathered is likely to be most accurate could contribute to a more targeted and efficient use of treatment resources.

### Study limitations

There are limitations to this study. As already noted, there is no clear reference standard to determine which assessment (preintervention or postintervention) is more accurate; that is, there are no biomarkers or collateral reports available. Additionally, we assessed drug use with an item that encompassed the full range of drug classes rather than separating them. Future research could profitably explore client answers about specific drug types. Fidelity checks were not included in either of these studies; this limits our ability to draw conclusions about differences – or lack thereof – between the two conditions in Study 2. Further investigation should include fidelity checks to allow for clearer conclusions about the effects of a motivational intervention. Finally, all instruments were self-administered. It may be that interviewer administered instruments might have increased participant comprehension, particularly in those with low education levels, producing different results. Conversely, it may be that the presence of an interviewer would alter the participants’ reports of use. Future research might profitably assess the differences these methods might make with participants.

## Competing interests

The authors are all employed by Prevention Research Institute (PRI), the private nonprofit organization who funded this study. PRI also created and distributes the PRIME For Life® program discussed in this study.

## Authors’ contributions

DR contributed to the writing and editing of this manuscript, providing primary oversight over all aspects of its preparation, conceptual basis, purpose, and interpretation. BB contributed to the conceptual underpinnings guiding the study, developed, performed and supervised data analysis, and participated in the text preparation. MN oversaw data collection, entry, and preparation. PS performed the statistical analysis, developed figures and tables, and contributed to text preparation and editing. MN and RD originally conceived the study, developed measures, and participated in text preparation and editing. All authors read and approved the final manuscript.
